# Phylogenetic comparative assembly

**DOI:** 10.1186/1748-7188-5-3

**Published:** 2010-01-04

**Authors:** Peter Husemann, Jens Stoye

**Affiliations:** 1 AG Genominformatik, Technische Fakultät, Bielefeld University, Germany; 2 International Graduate School in Bioinformatics and Genome Research, Bielefeld University, Germany; 3 Institute for Bioinformatics, Center for Biotechnology (CeBiTec), Bielefeld University, Germany

## Abstract

**Background:**

Recent high throughput sequencing technologies are capable of generating a huge amount of data for bacterial genome sequencing projects. Although current sequence assemblers successfully merge the overlapping reads, often several contigs remain which cannot be assembled any further. It is still costly and time consuming to close all the gaps in order to acquire the whole genomic sequence.

**Results:**

Here we propose an algorithm that takes several related genomes and their phylogenetic relationships into account to create a graph that contains the likelihood for each pair of contigs to be adjacent.

Subsequently, this graph can be used to compute a layout graph that shows the most promising contig adjacencies in order to aid biologists in finishing the complete genomic sequence. The layout graph shows unique contig orderings where possible, and the best alternatives where necessary.

**Conclusions:**

Our new algorithm for contig ordering uses sequence similarity as well as phylogenetic information to estimate adjacencies of contigs. An evaluation of our implementation shows that it performs better than recent approaches while being much faster at the same time.

## Background

Today the nucleotide sequences of many genomes are known. In the first genome projects, the process of obtaining the DNA sequence by multi-step clone-by-clone sequencing approaches was costly and tedious. Nowadays, the most common approach for de-novo genome sequencing is *whole genome shotgun sequencing *[1,2]. Here, the genome is fragmented randomly into small parts. Each of these fragments is sequenced, for example, with recent high throughput methods [3,4]. In the next step, overlapping reads are merged with an *assembler *software into a contiguous string. However, instead of the desired one sequence of the whole genome, often many *contigs *remain, separated by gaps. The main reasons for these gaps are lost fragments in the fragmentation phase and repeating sequences in the genome. In a process called *scaffolding*, the relative order of the contigs as well as the size of the gaps between them is estimated. In a subsequent *finishing *phase the gaps between the contigs are closed with a procedure called *primer walking*. For the ends of two estimated adjacent contigs, specific primer sequences have to be designed that function as start points for two polymerase chain reactions (PCRs) for Sanger sequencing [5]. These PCRs ideally run towards each other until the sequences overlap. To close a gap completely, new primer pairs have to be generated again and again since the maximum read length for Sanger sequencing is restricted. This makes the process expensive and work intensive. It is thus advisable to reduce the pairs of contigs that have to be considered. Given *n *contigs and no further information about their order, there are (*n*^2^) possibilities to apply primer walking. If the order is known, it suffices to do (*n*) primer walks to fill the gaps.

An algorithm that estimates a reasonable order for the contigs is thus a good help for sequencing projects. The estimation is usually based on the sequences of closely related species that are assumed to have a high degree of synteny. A few tools have been developed which use one or several related reference genomes to devise an ordering of the contigs: Projector2 [6], for example, is a web service that maps contig ends on a template genome using BLAST [7] or BLAT [8]. Features of Projector2 are an optional repeat masking for contig and template sequences, a visualization of the mapping, and an automated primer-design step for gap-closing purposes. Prior to the automated primer design, difficult regions for primer walking are removed, like, for example, repetitive sequences (phage DNA, IS elements or gene duplications) or sequences with unbalanced GC content. The program OSLay [9] takes a set of BLAST matches between the contigs and a reference sequence or scaffold and computes from this a layout for the contigs. The algorithm minimizes the height differences of so-called local diagonal extensions, which are basically matches from the border of a contig to the reference sequence. The resulting layout is visualized and can be imported into a Consed [10] project to aid gap-closure. Zhao et al. [11] present a method to find an ordering for a set of contigs using several related sequences as references. For each reference genome a *fitness matrix *is computed giving distances between the contigs, based on their BLAST matches. All matrices are combined into a single fitness matrix to search an optimal path of contig connections with their heuristic PGA (pheromone trail-based genetic algorithm).

Our work in this field commenced when analyzing data from in-house sequencing projects for different species of the *Corynebacteria *genus. We observed several aspects making it hard to find an ordering of the contigs. Zhao et al. [11] show that poor sequence coverage can be overcome by using multiple reference genomes, but problematic for this approach are major rearrangements in the genomic sequences of more distantly related species. Another challenge are repeating regions in the sequence of the newly sequenced genome. We developed an algorithm that uses the information of all similar regions between a set of contigs and several related reference genomes to estimate an ordering of the contigs. The novel idea is to incorporate the phylogenetic distance of the species in order to alleviate the impact of rearrangements to the ordering. While generating one 'optimal' order of the contigs is the predominant approach to aid the closure of gaps, we propose a more flexible format to describe contig adjacencies that is also capable of dealing with repeating contigs.

The algorithm we present here is based on a simple data structure, the *contig adjacency graph*, that is introduced in the next section. There we also give an optimal solution for finding a linear ordering of the contigs using this graph. However, a linear ordering is not sufficient to reflect all relations of real contig data. Therefore we propose a heuristic by which the most promising, but not necessarily unique, adjacencies are revealed in a *layout graph*. In section 'Results and discussion' we show the evaluation of applying our method to real sequencing data and compare the results with those obtained by PGA.

## Methods

A natural strategy to devise an 'optimal' linear ordering of the contigs, based on one or several related reference genomes, works in three steps: At first, all similar regions between the contigs and each reference genome are determined. Then a graph is created, containing edge weights that reflect the neighborhood of the contigs. In the last step a weight maximizing path through the graph is calculated, which defines the desired order of the contigs. In the following, we describe these three steps in more detail. In particular, in Section 'Contig adjacency graph', we define a novel edge weight function that incorporates the phylogenetic distance of the involved species.

### Matching contigs against a reference

Let Σ ={*A*, *C*, *G*, *T*} be the alphabet of nucleotides. We denote by Σ* the set of all finite strings over Σ, by |*s*|:= ℓ the *length *of string *s *= *s*_1 _... *s*_ℓ_, and by *s *[*i*, *j*]:= *s*_*i*_... *s*_*j *_with 1 ≤ *i *≤ *j *≤ ℓ the *substring *of *s *that starts at position *i *and ends at position *j*. Suppose we are given a set of contigs  ={*c*_1_, ..., *c*_*n*_}, *c*_*i *_∈ Σ*, and a set of already finished reference genomes ℛ ={*g*_1_, ..., *g*_*m*_}, *g*_*r *_∈ Σ*. The relation of the reference genomes is given by a phylogenetic tree  that contains the evolutionary distances of the species. Note that the tree can be generated even if some genomes are not completely assembled yet, for example from 16S-rRNAs. To infer information about the order and orientation of the contigs, these are mapped onto each reference genome by calculating local alignments. Let *s *= *c *[*s*_*b*_, *s*_*e*_] be a substring of contig *c *and *t *= *g *[*t*_*b*_, *t*_*e*_] be a substring of reference genome *g*. The tuple *m *= ((*s*_*b*_, *s*_*e*_), (*t*_*b*_, *t*_*e*_)) is called a *matching region *or simply *match *if *s *and *t *share sufficient sequence similarity. The *length *of a match, |*m*|:= *t*_*e*_-*t*_*b *_+1, is defined as the length of the covered substring in the reference genome. Sufficient sequence similarity could be defined, for example, by a BLAST hit of significance above a user-defined threshold. Another possibility, that we employ in our implementation, is to use the *swift *algorithm [12] for matching. It utilizes a *q*-gram index and provides for each match *m *the number of exactly matching *q*-grams, denoted as qhits(*m*), which can be used as a quality estimation for that match. Note that each contig can have several matches on a reference genome. For *s*_*b*_*> s*_*e *_we define *c *[*s*_*b*_, *s*_*e*_] to be the reverse complement of *c *[*s*_*e*_, *s*_*b*_] and call *m *a *reverse match*. Further we assume w.l.o.g. that *t*_*b*_*< t*_*e *_for all *g *[*t*_*b*_, *t*_*e*_], otherwise we can replace the involved contig sequence by its reverse complement. For brevity of notation,  denotes a match between contig *c*_*i *_∈  and reference genome *g*_*r *_∈ ℛ, and  denotes the (possibly empty) set of all such matches.

**Figure 1:**
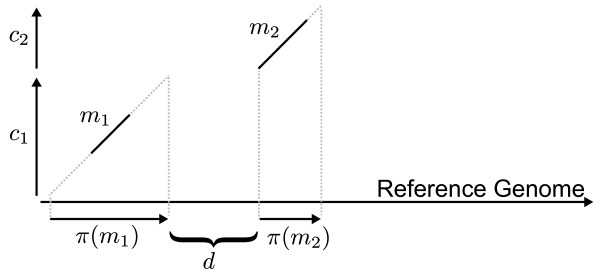
**Projection of a match**. Projections *π *(*m*_1_) and *π *(*m*_2_) of the contigs *c*_1 _and *c*_2 _based on their matches *m*_1 _and *m*_2_. The distance *d *reflects the displacement of the projections.

Each match  = ((*s*_*b*_, *s*_*e*_), (*t*_*b*_, *t*_*e*_)) implies a projection of the contig *c*_*i *_onto the reference genome *g*_*r *_. The *projected contig π *() = ((*t*_*b*_- *s*_*b*_), (*t*_*e*_+ |*c*_*i*_| - *s*_*e*_)) refers to the implied pair of index positions on *g*_*r *_. For reverse complement matches, the projection can be defined similarly. Figure 1 shows an example of two projected contigs as well as their distance, which will be defined next.

The *distance *of two projected contigs *π *(*m*) = (*t*_*b*_, *t*_*e*_) and *π *(*m'*) = () is defined as follows:

If the matches refer to different reference genomes, the distance of their projections is undefined. Note that the term distance is used here in the sense of 'displacement', *d *is *not *a metric in the mathematical sense. For example, *d *is negative if the projected contigs overlap.

### Contig adjacency graph

In the following we define the edge-weighted *contig adjacency graph * = (*V*, *E*) that contains for each contig *c*_*i *_∈  two vertices: *l*_*i *_as the *left connector *and *r*_*i *_as the *right connector *of contig *c*_*i*_. The set of vertices *V *is then defined as *V *= *L *∪ *R*, where *L *={*l*_1_, ..., *l*_*n*_} and *R *= {*r*_1_, ..., *r*_*n*_}.

The graph  is fully connected: *E *= (). We split these edges into two subsets: the *intra contig edges I *={{*l*_1_, *r*_1_}, ..., {*l*_*n*_, *r*_*n*_}} which connect for each contig its left and its right connector; and the set of *adjacency edges A *= *E*\*I *that connect the contigs among each other.

**Figure 2:**
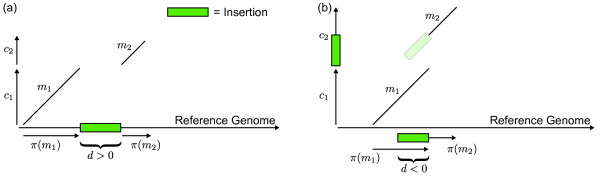
**Insertion distance**. (a) An insertion in the reference genome leads to a positive distance, whereas (b) an insertion in a contig leads to a negative distance.

Now we define a weight function for the edges. For each intra contig edge *e *∈ *I *we set the weight *w*(*e*) = 0. For the remaining edges let *e *= {*v*_*i*_, *v*_*j*_} ∈ *A *with *v*_*i *_∈ {*l*_*i*_, *r*_*i*_} and *v*_*j*_*∈ *{*l*_*j*_, *r*_*j*_} be an adjacency between contigs *c*_*i *_and *c*_*j*_. Then the weight of this adjacency edge is defined as

where the (symmetric) function *w*_*r*_(*v*_*i*_, *v*_*j*_) defines a likelihood score for the contigs *c*_*i *_and *c*_*j *_being adjacent, with respect to their connectors *v*_*i *_and *v*_*j *_. Each score *w*_*r *_is based on the matches to reference *g*_*r *_and employs the phylogenetic distance  between the contig species and the reference genome species as a weight factor:

where *d *is the distance between the projected contigs and *s*(*d*, ) is a suitably defined scoring function. In order to define *s *we will give some further biological motivations. The scoring function *s *models the likelihood that two contigs are adjacent based on the distance *d *of their projected contigs. Projected contigs that are not adjacent have a high distance and should obtain a low score. Adjacent contigs should gain a high score for usually having a distance close to zero. However, the distance of two projected contigs can reach positive values due to insertions in the reference's genome. Similarly, the distances can be negative if the projections overlap, which is the case if there are insertions in the newly sequenced genome. Both cases can be seen in Figure 2. Note that an insertion in the one genome corresponds to a deletion in the other.

A second important aspect that is included in our model are rearrangements between the related species, which can lead to misleading adjacencies of projected contigs. Assuming that between closer related species less rearrangements have taken place, we use the phylogenetic tree distance  to weight the match information.

To model the two mentioned considerations, we use a Gaussian distribution with an expected value of zero:

where *s *is the standard deviation for the size of deletions or insertions. A higher tree distance  allows larger insertions and deletions, but scores the reliability of the matches to more distantly related genomes to a lesser degree.

However, this model neglects the fact that in the fragmentation phase, for example in parallel pyrosequencing, often fragments disappear, such that there are no reads for this fragment. If a fragment is not sequenced, the same situation arises as if there is an insertion into the reference genome, which causes positive distances. To include this detail we use two superimposed Gaussian distributions for the scoring. The first distribution models insertions into the contigs and into the reference genome, the second models lost fragments during sequence assembly. The influence of each model is determined by a weighting factor *φ*:(1)

The expected value *μ *of the second Gaussian distribution is equal to the average size of the lost fragments.

The standard deviations *σ*_1 _and *σ*_2 _can be estimated from sequencing projects.

### Finding a tour through the graph

The contig adjacency graph with the described edge weights can be used to find a linear ordering of the contigs. This can be achieved by computing a tour through the graph that incorporates all contigs and maximizes the total weight. With minor enhancements of the graph, this becomes equivalent to finding a shortest Hamiltonian cycle.

The modifications are as follows: At first all edge weights have to be converted to distances. This is done by replacing each edge weight *w *by *m - w *where *m *is the maximum weight in the graph. To ensure that each contig is incorporated exactly once, and only in one direction, we add an intermediate node between the left and the right connector of each contig. The modified graph is then defined as  = (V', E') with *V' *= *V *∪ {*v*_*i*_| 1 ≤ *i *≤ *n*} and *E' *= *A *∪ {{*l*_*i*_, *v*_*i*_}, {*v*_*i*_, *r*_*i*_}| 1 ≤ *i *≤ *n*}. The distance of all edges that lead to an intermediate node *v*_*i *_is set to 0. It is easy to see that a shortest Hamiltonian cycle in the modified graph defines an ordering as well as the orientation of all contigs, and thus any TSP algorithm can be used to find an optimal linear layout of the contigs.

### Fast adjacency discovery algorithm

As described in the previous section, a linear ordering of the contigs, which is optimal with respect to the adjacency edge weights, can be computed using a suitable optimization algorithm. However, our analysis of real data in the results section shows that a linear order of the contigs is not necessarily possible, mainly due to arbitrary placement of repeated or rearranged regions. A method that provides a unique layout where possible, but also points out alternative solutions where necessary, may be more useful in practice. We present an approach following this overall strategy in this section.

The basis of our algorithm is a greedy heuristic for the TSP, known as the *multi-fragment heuristic *[13], that proceeds as follows: First the edges of the graph are sorted by increasing distance and then added in this order into an initially empty set of path fragments. Whenever an involved node would exceed the maximal degree of two, or if a path fragment would create a cycle, the edge is skipped. The only exception to the latter is the final Hamiltonian cycle of length *n*.

This *best connection first *procedure creates multiple low distance path fragments which are merged sooner or later. We chose this approach because it seems natural to incorporate those adjacencies first into an ordering that are most promising to be investigated for gap closure.

**Figure 3:**
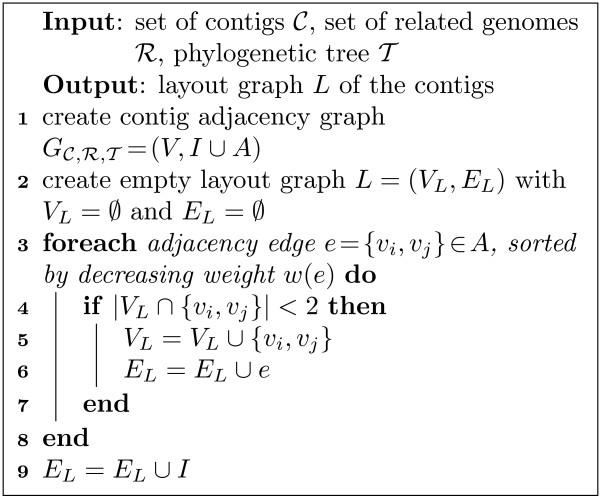
**Contig adjacency discovery algorithm**. Heuristic to compute the layout graph which shows the most promising contig adjacencies.

As already indicated, repeating or rearranged regions may prohibit an unambiguous linear ordering of the contigs. Repeating contigs create cycles in a possible path, and rearrangements can lead to conflicting adjacencies of a contig. To model both, we relax the constraints of the multi-fragment heuristic: First, we do not check for cycles, which permits repeating contigs to be incorporated adequately. Secondly, when inserting an edge, we allow one of the incident nodes, but not both, to exceed the degree of two, which allows to also include conflicting information into our layout. The result of this modified heuristic is a subgraph of the contig adjacency graph *L *⊂  that we call the *layout graph*. The algorithm to generate the layout graph is formally described in Figure 3. Note that the resulting layout graph is not necessarily connected.

The layout graph can be analyzed to make assumptions about repeating contigs and rearrangements. Conflicting edges can give hints about these two problems, whereas the information about unambiguously incorporated contigs can be used to generate primer pairs for gap closure. Displaying also the ambiguities allows to investigate the conflicting connections further. Instead of pinning the result down to a single, possibly wrong, order of the contigs we prefer to output the best possibilities. Nonetheless it should be kept in mind that rearrangements can cause seemingly good adjacencies that do not belong to a correct layout.

### Integrating additional information

In sequencing projects often additional information occurs which can be helpful for the scaffolding of a genome. After introducing two of such information types, we outline how they may be incorporated into our approach.

1. Recent protocols for parallel pyrosequencing produce *mate pair information*. Fragments of a defined size are sequenced from both ends thus providing the approximate distance as well as the orientation of both reads relative to each other. This information simplifies the assembly and sometimes indicates adjacencies of contigs.

2. After an initial assembly of the reads, often *fosmid libraries *are employed to accomplish the finishing of a genome. Fragments of the genome with a size between 35 and 40 kb are used as inserts for the fosmids in order to sequence the ends of each inserted fragment. If those end sequences can be mapped to different contigs, it is possible to infer the distance and orientation of contigs towards each other. Fosmid libraries have the additional advantage that they can be used for primer walking even if the gaps between the contigs exceed the usual size to do primer walking on the genome. But of course this advantage is paid for with a high amount of work to create the library.

The information from mate pairs, fosmid libraries or on a larger scale even radiation hybrid maps can be included into our approach by modifying the weights of the computed contig adjacency graph. This influences also the predicted adjacencies in the layout graph which is the outcome of our algorithm. If expert information indicates that two contigs are not adjacent, it suffices to set the appropriate edge weight to zero. This contig connection will not occur in the result afterwards. On the contrary, if for example fosmid end sequencing shows that two contigs are adjacent and quite close, the incorporation of that edge into the layout graph can be forced by setting the corresponding edge weight to the maximum weight of the graph.

## Results and discussion

### Datasets

To evaluate our proposed method, we prepared three datasets, each consisting of a set of contigs to be layouted and a set of reference genomes which are related to the contig's genome. From sequencing projects conducted at Bielefeld University, we obtained the contig sequences for three genomes of the *Corynebacteria *genus: *C. aurimucosum *(NC_012590), *C. urealyticum *[14], and *C. kroppenstedtii *[15]. The complete genomes of these species have already been finished and are available from NCBI [16,17]. This enables us to compute a *reference order *for each set of contigs that serves as a 'standard of truth'. The reference order was devised by mapping the contigs onto their corresponding finished genome and placing each contig on that region where it gained the most matches. We would like to note that such a reference order is not necessarily unique since contigs often contain or even consist of repeating regions which map non-uniquely to multiple locations. On the contrary, some of the contigs do not even match at all and could for this reason not be included in the reference order: Five out of originally 113 contigs were affected in the *C. aurimucosum *genome. Two of them are very small (241 and 304 bases, respectively) but the other three have a considerable size of 28 kb in total. The explanation for the latter not matching is that the sequences belong to the *C. aurimucosum *plasmid pET44827. On the finished *C. kroppenstedtii *genome, two very small contigs (118 and 222 bases) out of 11 could not be matched. For *C. urealyticum *we used all contigs larger than 500 bases since many smaller contigs could not be placed appropriately. This way 154 of originally 223 contigs with a total size of 22 kb were not used which equals less than 0.01% of the finished genome. Moreover, the N50 contig size, which is a more robust measure than the mean or median to characterize the contig's size distribution, stays the same for the reduced set. In all following experiments the contig sets were composed of only those contigs that also appear in the reference order. The contig sets together with some important properties are shown in Table 1.

**Table 1:** Contig sets

Contig Organism	# Contigs	Total Length	N50 Contig Size
*C. aurimucosum *ATCC 700975	108	2 716 204 bp	96 704 bp
*C. kroppenstedtii *DSM 44385	9	2 434 935 bp	546 376 bp
*C. urealyticum *DSM 7109	69	2 294 755 bp	86 391 bp

The three contig sets that were used to evaluate our algorithm. All corresponding genomes have already been finished. The N50 contig size gives the size of the largest contig such that at least half of the total size is covered by contigs larger than that contig. This measure is more robust than the mean or median if many small contigs exist.

**Figure 4:**
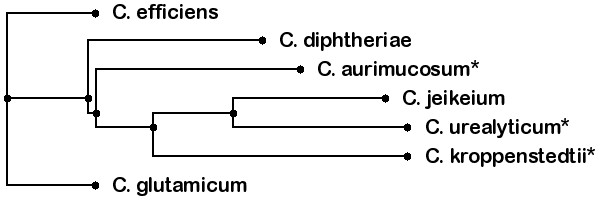
**Phylogenetic tree**. Phylogenetic tree of the employed Corynebacteria. For all species marked with an asterisk (*) the underlying contig data were available. The tree was calculated with EDGAR [18], the image was generated with PHY.FI [23].

**Figure 5:**
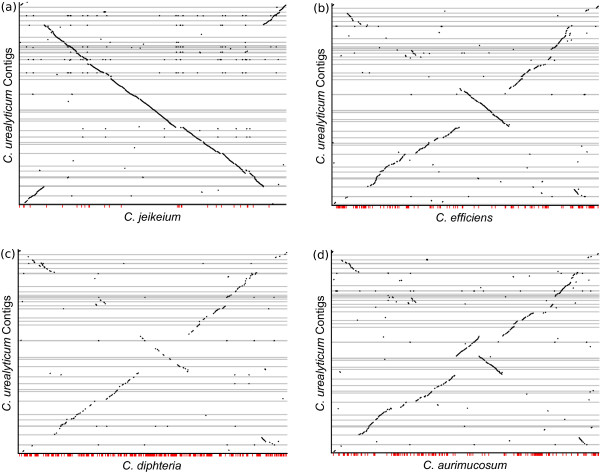
**Synteny plots**. Pairwise synteny plots of the contigs of *C. urealyticum *and four chosen complete genomes of the *Corynebacteria *genus. The contigs are stacked on the vertical axis in reference order, separated by horizontal lines. The ticks below each synteny plot indicate uncovered regions.

As reference genomes for ordering the contigs, the three above mentioned finished genomes were used and were extended by choosing four more publicly available *Corynebacteria *genomes, *C. diphtheriae*, *C. efficiens*, *C. glutamicum*, and *C. jeikeium*, that we downloaded from NCBI. The complete set of reference genomes, including their accession numbers, is shown in Table 2. Obviously, whenever a genome is to be reconstructed from its set of contigs, this genome is removed from the dataset of reference genomes. For our algorithm the phylogenetic relationship between the species plays an important role. Figure 4 shows the evolutionary tree of all employed genomes. The tree was generated with the EDGAR framework [18] applying Neighbor Joining [19] to a set of *core *genes. As a more detailed illustration for the varying degree of rearrangements and synteny between the employed species, Figure 5 shows four example synteny plots for the contigs of *C. urealyticum*. While Figure 5(a) shows a high degree of synteny and only few rearrangements to the *C. jeikeium *genome, Figure 5(d) shows low synteny combined with many major rearrangements in the *C. aurimucosum *genome. Figures 5(b) and 5(c) show similar rearrangements but differing levels of synteny with respect to the genomes of *C. efficiens *and *C. diphtheriae*. It is clearly observable that due to rearrangements a mapping of the contigs on the displayed related genomes would provide incorrect adjacencies of some contigs.

**Table 2:** Reference genomes

Reference Organism	Sequence Length	**Accession Nr**.
*C. aurimucosum *ATCC 700975	2 790 189 bp	NC_012590
*C. diphtheriae *NCTC 13129	2 488 635 bp	NC_002935
*C. efficiens *YS-314	3 147 090 bp	NC_004369
*C. glutamicum *ATCC 13032	3 282 708 bp	NC_006958
*C. jeikeium *K411	2 462 499 bp	NC_007164
*C. jeikeium *K411 plasmid pKW4	14 323 bp	NC_003080
*C. kroppenstedtii *DSM 44385	2 446 804 bp	NC_012704
*C. urealyticum *DSM 7109	2 369 219 bp	NC_010545

Overview of the reference sequences of the *Corynebacteria *genus that were employed for our study.

### Experimental setup

In the following we address the outcome of three different experiments: At first we used PGA [11] to order the contigs, given the finished genome as reference. Secondly, we applied OSLay [9] and Projector2 [6] to find an ordering of the contigs, each time with the closest genome as reference sequence. Finally, as an evaluation of our proposed method, we compare the performance of PGA and our implementation *treecat *using all reference genomes except for the one to be finished.

In each experiment, the mentioned programs were run to devise an ordering of the contigs. The output was then compared to the reference order. We counted all connections which also occur in the corresponding reference order as *true positives *(TP), all others as *false positives *(FP). From these values we calculated the *sensitivity *(also called *true positive rate*, , where P is the number of connections in the reference order) as well as the *precision *(also called *positive predictive value*, ). All four values are given in the result tables for each experiment. For PGA, which is a randomized algorithm, the results were often varying, so we give the mean values for applying the program 20 times. Additionally the best result, which yielded the highest TP while having the lowest FP, is given in parentheses.

### Assessing the difficulty of comparative assembly

#### Uniqueness of a linear order

Our first experiment serves as a demonstration how difficult it is to order the contigs of the given datasets. PGA was applied on each contig set with only one reference, the already finished genome of the contigs, which ought to provide the 'perfect' information.

A run of PGA proceeds as follows: First BLAST is used to match the contigs on each given genome. After that PGA computes five paths for the contigs that optimize a *fitness matrix *which is comparable to our contig adjacency graph. For that purpose a genetic algorithm is used, possibly giving different connections with each run. The connections of all five paths are included into the result together with a weight giving the percentage how often this connection occurred.

**Figure 6:**
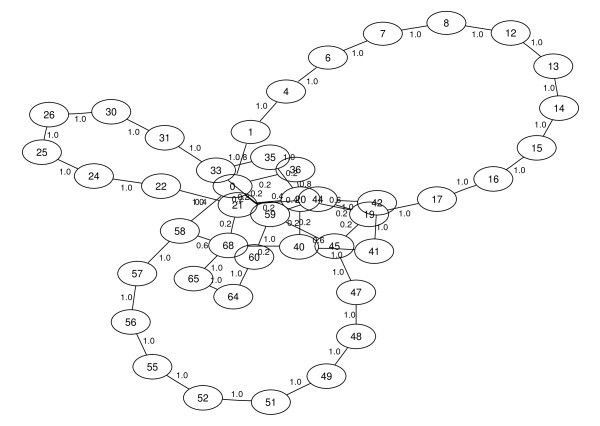
**PGA with perfect reference**. *C. urealyticum *contig connections generated by PGA when using the finished genome as reference sequence. Here, the best result (25 TP, 31 FP) achieved in 20 runs is displayed. The contig nodes are numbered in reference order.

The results of this experiment are shown in Table 3. Both sensitivity and precision are comparably low for all datasets. We see the reason for this mainly in repeating sequences flanking the contigs. Sometimes almost the whole sequence of a contig is repeating which leads to cycles in a potential path that orders the contigs. This can clearly be observed in Figure 6 which visualizes the contig connections that PGA predicts for the example of *C. urealyticum*. The node labels in this graph are the ranks of the corresponding contigs with respect to the reference order of the contigs. The correct path should therefore be 0, 1, ..., 68. Some nodes are missing in this graph since PGA filters all contigs of length less than 3 500 bases. The edge labels give the percentage how often a connection occurs in the five paths. In this example it is observable that there are loops of connections where the contigs are almost uniquely orderable but there also occur parts where such a linear order can not be achieved. This experiment shows that it is not feasible to generate a linear ordering if repeating regions would create cycles in a potential path. If the contig adjacencies are not unique, then, to our opinion, it would be better to show the most probable alternatives instead of relying on a linear path. This, however increases the false positive rate of results created that way. In fact PGA does show alternatives by combining the results of five paths but each path corresponds still to a linear ordering. One has to decide what is more valuable: A single linear ordering which could also be wrong or the display of possible alternatives with the risk of producing some false positives. We believe that the latter pays off when trying to manually close the gaps during genome finishing.

**Table 3:** PGA results - Perfect reference

	PGA			
**Organism**	**TP**	**FP**	**TPR**	**PPV**

*C. aurimucosum*	19.3 (20)	44.2 (40)	0.18 (0.19)	0.30 (0.33)
*C. kroppenstedtii*	3.0 (3)	3.0 (3)	0.33 (0.33)	0.50 (0.50)
*C. urealyticum*	24.2 (25)	33.4 (31)	0.35 (0.36)	0.42 (0.45)

Results of applying PGA using the already finished genome as reference sequence. The values are averaged over 20 times applying PGA. The best result of these runs is given in parentheses.

#### Single reference based ordering

The second experiment was designed to underline that the incorporation of several related genomes is favorable to the use of only a single reference sequence. Therefore, we applied OSLay [9] and Projector2 [6] on our datasets using the closest phylogenetic neighbor as reference sequence.

To generate the OSLay results, the contigs were mapped using nucmer from the MUMMER package [20]. We used OSLay then to compute an optimal syntenic layout of the contigs using the standard parameters of the implementation. The adjacencies were finally extracted from the supercontigsList.x.txt files. The Projector2 results were generated using its web-service with standard parameters. The matching was performed by running BLAT on the server.

The results for OSLay and Projector2 are shown in Table 4. Both programs do not predict many connections that also occur in the reference order. Although a direct comparison is not fair we will see in the next experiment that the use of multiple related genomes as reference sequences improves the resulting layouts.

**Table 4:** OSLay and Projector2 results

		OSLay					Projector2			
				
Organism	Closest Reference	TP	FP	TPR	PPV		TP	FP	TPR	PPV
*C. aurimucosum*	*C. glutamicum*	0	1	0.00	0.00		10	20	0.09	0.33
*C. kroppenstedtii*	*C. jeikeium*	0	0	0.00	*undef*.		1	2	0.11	0.33
*C. urealyticum*	*C. jeikeium*	6	6	0.09	0.50		8	18	0.12	0.31

Results of OSLay and Projector2 using the phylogenetically closest genome as reference sequence.

### Evaluation

#### Implementation treecat

We implemented our proposed algorithm in Java. The software *treecat *(tree based contig arrangement tool) contains a re-implementation of the fast local alignment algorithm *swift *[12], the contig adjacency graph creation, a branch and bound exact TSP algorithm, and the fast layout graph heuristic described in section 'Fast adjacency discovery algorithm'. The software is open source (GPL) and available within the *Comparative Genomics - Contig Arrangement Toolsuite *(cg-cat, http://bibiserv.techfak.uni-bielefeld.de/cg-cat) on the Bielefeld Bioinformatics Server (BiBiServ). Input to *treecat *are the FASTA [21] sequences of the contigs and of the related references as well as a phylogenetic tree in Newick format. Each reference can consist of several sequences, for example several chromosomes. When the algorithm is run, first all matches from the contigs to each reference are computed. For the following results, matches were considered to have a minimal length of 64 bases and a maximum error rate of 8%. The matches are cached which allows a visualization like in Figure 5 and avoids a new computation if subsequent steps are re-run with different parameters. As the second step, after the matching, the contig adjacency graph is constructed as defined in the Methods section. The following (empirically estimated) parameters were used for the scoring function (1) to compute the results: The standard deviation of the insertion/deletion size was set to *σ*_1 _= 10 000 bases and the expected lost fragment size to *μ *= 2 000 bases with a standard deviation of *σ*_2 _= 1 000 bases. The lost fragment weighting factor *φ *was set to 0.1. In the last step, the computed adjacency graph is used to devise the contig layout graph which can then be visualized with the open source software package GraphViz [22].

#### Comparison of PGA and treecat

In this experiment we applied our new algorithm to the three evaluation datasets and compared the results to the output of PGA. All sequences of Table 2, except the genome of the contigs to be layouted, served as references to find a layout for one of the contig sets in Table 1. PGA was run using the standard parameters given in [11], for *treecat *the parameters were used as stated above. The results of this comparison are listed in Table 5 and the running times of both programs, for matching and layouting, are shown in Table 6. The comparison shows that our method achieves in general better results than PGA, even compared to the best PGA result out of 20 runs, while being much faster.

**Table 5:** PGA and treecat results using multiple references

	PGA					*treecat*			
			
Organism	TP	FP	TPR	PPV		TP	FP	TPR	PPV
*C. aurimucosum*	14.5 (16)	66.5 (70)	0.13 (0.15)	0.18 (0.19)		**17**	**66**	**0.16**	**0.20**
*C. kroppenstedtii*	2.0 (2)	**4.0 **(**4**)	0.22 (0.22)	**0.33 **(**0.33**)		**3**	6	**0.33**	**0.33**
*C. urealyticum*	20.9 (25)	72.5 (76)	0.30 (0.36)	0.22 (0.25)		**27**	**70**	**0.39**	**0.28**

Evaluation of applying PGA and *treecat *for ordering the sets of contigs with the help of the remaining genomes as references. PGA's results are averaged over 20 runs, the best run (highest TP with lowest FP) is given in parentheses. The overal best result is printed in bold face.

**Table 6:** PGA and treecat results using multiple references: Times

	PGA			*treecat*	
			
Organism	Matching	Layouting		Matching	Layouting
*C. aurimucosum*	436.2 s	184.1 s		**104.4 **s	**0.9 **s
*C. kroppenstedtii*	208.4 s	25.6 s		**83.4 **s	**0.4 **s
*C. urealyticum*	458.8 s	161.5 s		**100.0 **s	**1.2 **s

Running times for PGA and *treecat *to generate the results of Table 5. The matching for PGA, done with BLAST, was measured only once, the times for layouting are the mean of 20 runs. The times for treecat are the mean of three runs. All times are given in seconds for performing the experiments on a sparcv9 processor operating at 750 MHz under Solaris.

**Figure 7:**
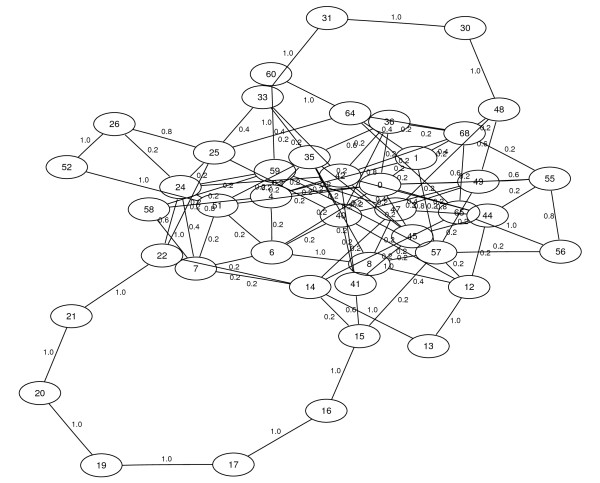
**PGA with multiple references**. The best result (25 TP, 76 FP) PGA generated in 20 runs for ordering the *C. urealyticum *contigs when using all other genomes as reference sequences. The contig nodes are numbered in reference order.

**Figure 8:**
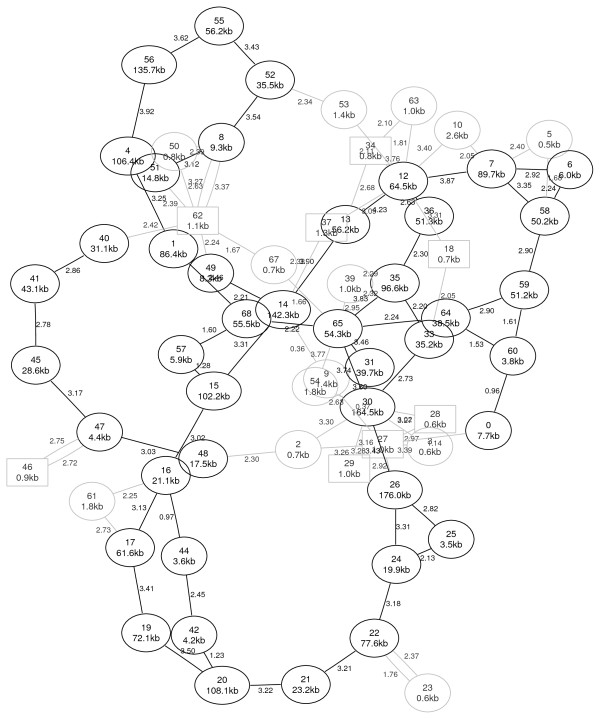
**treecat with multiple references**. *C. urealyticum *contig connections generated by *treecat *when using all other genomes as reference sequences. The contig nodes are numbered in reference order. Contigs smaller than 3.5 kb have gray nodes, repeating contigs for which at least 95% of the sequence occurs more than once on a reference genome have rectangular nodes. Edge weights are given in logarithmic scale.

As an illustration of the results, Figures 7 and 8 show the resulting graphs of PGA and *treecat*, respectively, for the example of the *C. urealyticum *contigs. In both graphs the number of a node is again the rank of the corresponding contig with respect to the reference order. In Figure 8 some additional information about the size and the repetitiveness of the contigs is given. The second line of a node indicates the size of a contig in kb, contigs smaller than 3.5 kb are drawn in gray. Contigs of which more than 95% of the sequence is repeating on at least one reference genome have a rectangular node. The edge weights in Figure 8 are the weights calculated with the scoring function (1) in logarithmic scale.

Comparing the graphs of both programs visually gives the impression that the *treecat *result is a little less cluttered. This impression becomes even stronger if all small nodes in gray are ignored.

There are a few details to mention when investigating the resulting graphs for ordering the *C. urealyticum *contigs. PGA's graph contains a connection placing contig 26 next to contig 52 which is obviously incorrect. Our approach does not show this connection. Manual inspection shows that this is due to the evolutionary distances that we incorporate for the edge scoring since phylogenetically closer genomes do not contain this adjacency. This is further supported by the fact that the connection is also not present when PGA uses the 'perfect' reference, see Figure 6.

In our graph the connection from contig 4 to contig 56 as well as the connection from contig 7 to contig 58 has a high score but is not correct. This is explainable due to the big inversion, see Figure 5(a), in the *C. jeikeium *genome which is the next phylogenetic neighbor to *C. urealyticum *and therefore has a high influence on our result.

Further investigation of the *treecat *result in Figure 8 gives additional details: For the contigs 12 to 26, an almost unique path can be observed that orders most of the inner contigs. Repeating contigs like 27 or 62 show a star-like adjacency pattern which can be explained by different adjacencies of the corresponding repeat occurrences. However, there are also larger non-repeating contigs, like contig 12, that show such a star pattern. In this case several different repeating contigs neighbor this contig on different references. The graph shows a double connection 46 to 47, which can be explained by a repeating reverse complement match of a part of contig 46 that lies once before contig 47 and once behind it. This results in a high edge weight for the left connectors {*l*_46_, *l*_47_}, as well as the right connectors {*r*_46_, *r*_47_} of these contigs. Some connections, like 52 to 55, seem to imply missing contigs, but further investigation reveals that contigs 53 and 54 are partially repeating contigs which fit between the mentioned contigs but as well next to other contigs. There are adjacencies in the layout graph that have a high difference in the rank of the reference order. For example the connection from contig 16 to 44 seems erroneous but it can be explained by a 1.5 kb repeating substring of contig 44. Similar observations can be made in several other places.

## Conclusions

In this paper, we presented an algorithm that orders a set of contigs, given several related reference genomes and a phylogenetic tree of the involved species. In particular, we proposed a more flexible output for the ordering of contigs since our results demonstrate that for real world data the search for one linear optimal ordering of the contigs is not feasible. Consequently, our algorithm allows alternative connections in a layout which is necessary because of repeating regions and rearrangements between the species. Secondly, we introduced a novel scoring function for the contig adjacency estimation that is biologically motivated in two ways: It contains a sophisticated weighting scheme for the distances of projected contigs and it integrates the phylogenetic distances of the species to alleviate the effects caused by rearrangements. An evaluation of our algorithm shows that its implementation *treecat *is considerably faster than a recent approach from the literature while it is at the same time generating better results. We believe that with our approach of including phylogenetic information into the problem of contig layouting, we have gone one step further in using all available information for this important task within genome finishing.

Nevertheless, in sequencing projects, often additional information emerges which is not yet included in our approach. For example, information derived from mate pairs, fosmid libraries or radiation hybrid maps might give valuable hints on the orientation and the distance of contigs while not being biased by evolutionary events. Concerning the phylogenetic tree, rearrangements between the genomes were not predicted by the methods presented in this paper. This leads to ambiguous information for the ordering of contigs and thus to weak or misleading adjacency scores which need to be curated manually. A strategy for the discovery of rearrangements is thus desired in future work. Furthermore, due to horizontal gene transfer some regions of a genome can have different evolutionary histories than others. Detecting such regions and treating them in a special way might be advisable in an even more sophisticated approach.

## Competing interests

The authors declare that they have no competing interests.

## Authors' contributions

PH conceived the algorithm, implemented the software, performed the evaluation, and drafted the manuscript. JS supervised this work, provided the initial idea and contributed to the editing of the manuscript. Both authors read and approved the final manuscript.
